# A European Multi Lake Survey dataset of environmental variables, phytoplankton pigments and cyanotoxins

**DOI:** 10.1038/sdata.2018.226

**Published:** 2018-10-23

**Authors:** Evanthia Mantzouki, James Campbell, Emiel van Loon, Petra Visser, Iosif Konstantinou, Maria Antoniou, Grégory Giuliani, Danielle Machado-Vieira, Alinne Gurjão de Oliveira, Dubravka Špoljarić Maronić, Filip Stević, Tanja Žuna Pfeiffer, Itana Bokan Vucelić, Petar Žutinić, Marija Gligora Udovič, Anđelka Plenković-Moraj, Nikoletta Tsiarta, Luděk Bláha, Rodan Geriš, Markéta Fránková, Kirsten Seestern Christoffersen, Trine Perlt Warming, Tõnu Feldmann, Alo Laas, Kristel Panksep, Lea Tuvikene, Kersti Kangro, Kerstin Häggqvist, Pauliina Salmi, Lauri Arvola, Jutta Fastner, Dietmar Straile, Karl-Otto Rothhaupt, Jeremy Fonvielle, Hans-Peter Grossart, Christos Avagianos, Triantafyllos Kaloudis, Theodoros Triantis, Sevasti-Kiriaki Zervou, Anastasia Hiskia, Spyros Gkelis, Manthos Panou, Valerie McCarthy, Victor C. Perello, Ulrike Obertegger, Adriano Boscaini, Giovanna Flaim, Nico Salmaso, Leonardo Cerasino, Judita Koreivienė, Jūratė Karosienė, Jūratė Kasperovičienė, Ksenija Savadova, Irma Vitonytė, Sigrid Haande, Birger Skjelbred, Magdalena Grabowska, Maciej Karpowicz, Damian Chmura, Lidia Nawrocka, Justyna Kobos, Hanna Mazur-Marzec, Pablo Alcaraz-Párraga, Elżbieta Wilk-Woźniak, Wojciech Krztoń, Edward Walusiak, Ilona Gagala, Joana Mankiewicz-Boczek, Magdalena Toporowska, Barbara Pawlik-Skowronska, Michał Niedźwiecki, Wojciech Pęczuła, Agnieszka Napiórkowska-Krzebietke, Julita Dunalska, Justyna Sieńska, Daniel Szymański, Marek Kruk, Agnieszka Budzyńska, Ryszard Goldyn, Anna Kozak, Joanna Rosińska, Elżbieta Szeląg-Wasielewska, Piotr Domek, Natalia Jakubowska-Krepska, Kinga Kwasizur, Beata Messyasz, Aleksandra Pełechata, Mariusz Pełechaty, Mikolaj Kokocinski, Beata Madrecka, Iwona Kostrzewska-Szlakowska, Magdalena Frąk, Agnieszka Bańkowska-Sobczak, Michał Wasilewicz, Agnieszka Ochocka, Agnieszka Pasztaleniec, Iwona Jasser, Ana M. Antão-Geraldes, Manel Leira, Armand Hernández, Vitor Vasconcelos, João Morais, Micaela Vale, Pedro M. Raposeiro, Vítor Gonçalves, Boris Aleksovski, Svetislav Krstić, Hana Nemova, Iveta Drastichova, Lucia Chomova, Spela Remec-Rekar, Tina Elersek, Jordi Delgado-Martín, David García, Jose Luís Cereijo, Joan Gomà, Mari Carmen Trapote, Teresa Vegas-Vilarrúbia, Biel Obrador, Ana García-Murcia, Monserrat Real, Elvira Romans, Jordi Noguero-Ribes, David Parreño Duque, Elísabeth Fernández-Morán, Bárbara Úbeda, José Ángel Gálvez, Rafael Marcé, Núria Catalán, Carmen Pérez-Martínez, Eloísa Ramos-Rodríguez, Carmen Cillero-Castro, Enrique Moreno-Ostos, José María Blanco, Valeriano Rodríguez, Jorge Juan Montes-Pérez, Roberto L. Palomino, Estela Rodríguez-Pérez, Rafael Carballeira, Antonio Camacho, Antonio Picazo, Carlos Rochera, Anna C. Santamans, Carmen Ferriol, Susana Romo, Juan Miguel Soria, Lars-Anders Hansson, Pablo Urrutia-Cordero, Arda Özen, Andrea G. Bravo, Moritz Buck, William Colom-Montero, Kristiina Mustonen, Don Pierson, Yang Yang, Jolanda M. H. Verspagen, Lisette N. de Senerpont Domis, Laura Seelen, Sven Teurlincx, Yvon Verstijnen, Miquel Lürling, Valentini Maliaka, Elisabeth J. Faassen, Delphine Latour, Cayelan C. Carey, Hans W. Paerl, Andrea Torokne, Tünay Karan, Nilsun Demir, Meryem Beklioğlu, Nur Filiz, Eti E. Levi, Uğur Iskin, Gizem Bezirci, Ülkü Nihan Tavşanoğlu, Kemal Çelik, Koray Özhan, Nusret Karakaya, Mehmet Ali Turan Koçer, Mete Yilmaz, Faruk Maraşlıoğlu, Özden Fakioglu, Elif Neyran Soylu, Meral Apaydın Yağcı, Şakir Çınar, Kadir Çapkın, Abdulkadir Yağcı, Mehmet Cesur, Fuat Bilgin, Cafer Bulut, Rahmi Uysal, Latife Köker, Reyhan Akçaalan, Meriç Albay, Mehmet Tahir Alp, Korhan Özkan, Tuğba Ongun Sevindik, Hatice Tunca, Burçin Önem, Jessica Richardson, Christine Edwards, Victoria Bergkemper, Sarah O'Leary, Eilish Beirne, Hannah Cromie, Bastiaan W. Ibelings

**Affiliations:** 1 Department F.-A. Forel for Environmental and Aquatic Sciences, University of Geneva, 1205 Geneva, Switzerland; 2 Institute of Biology, Leiden University, 2333 BE Leiden, The Netherlands; 3 Department of Freshwater and Marine Ecology, University of Amsterdam, 1090 GE Amsterdam, Netherlands; 4 Department of Environmental Science and Technology, Cyprus University of Technology, 3036 Lemesos, Cyprus; 5 Institute for Environmental Sciences - GRID, University of Geneva, 1211 Geneva, Switzerland; 6 Departamento de Sistemática e Ecologia, Universidade Federal da Paraíba, 58059-970 Paraíba, Brasil; 7 Department of Biology, Josip Juraj Strossmayer University of Osijek, 31000 Osijek, Croatia; 8 Department for Ecotoxicology, Teaching Institute of Public Health of Primorje-Gorski Kotar County, 51000 Rijeka, Croatia; 9 Department of Biology, Faculty of Science, University of Zagreb, 10000 Zagreb, Croatia; 10 RECETOX, Faculty of Science, Masaryk University, 62500 Brno, Czech Republic; 11 Department of Hydrobiology, Morava Board Authority, 60200 Brno, Czech Republic; 12 Laboratory of Paleoecology, Institute of Botany, The Czech Academy of Sciences, 60200 Brno, Czech Republic; 13 Freshwater Biological Laboratory, Department of Biology, University of Copenhagen, 2100 Copenhagen, Denmark; 14 Institute of Agricultural and Environmental Sciences, Estonian University of Life Sciences, 51014 Tartu, Estonia; 15 Tartu Observatory, Faculty of Science and Technology, University of Tartu, 61602 Tartu, Estonia; 16 Department of Science and Engineering, Åbo Akademi University, 20520 Åbo, Finland; 17 Department of Biological and Environmental Science, University of Jyväskylä, 40014 Jyväskylä, Finland; 18 Lammi Biological Station, University of Helsinki, 16900 Lammi, Finland; 19 German Environment Agency, Unit Drinking Water Resources and Water Treatment, Corrensplatz 1, 14195 Berlin, Germany; 20 Department of Biology, Limnological Institute, University of Konstanz, 78464 Konstanz, Germany; 21 Department of Experimental Limnology, Leibniz Institute of Freshwater Ecology and Inland Fisheries, 16775 Stechlin, Germany; 22 Institute of Biochemistry and Biology, Potsdam University, 14469 Potsdam, Germany; 23 Water Quality Department, Athens Water Supply and Sewerage Company, 11146 Athens, Greece; 24 Institute of Nanoscience and Nanotechnology, National Center for Scientific Research «DEMOKRITOS», 15341 Attiki, Greece; 25 Department of Botany, Aristotle University of Thessaloniki, 54124 Thessaloniki, Greece; 26 Centre for Freshwater and Environmental Studies, Dundalk Institute of Technology, A91 K584 Dundalk, Ireland; 27 Department of Sustainable Ecosystems and Bioresources, Fondazione Edmund Mach, 38010 San Michele all’Adige, Italy; 28 Institute of Botany, Nature Research Centre, Vilnius 08412, Lithuania; 29 Department of Freshwater Ecology, Norwegian Institute for Water Research, 0349 Oslo, Norway; 30 Department of Hydrobiology, University of Bialystok, 15245 Bialystok, Poland; 31 Institute of Environmental Protection and Engineering, University of Bielsko-Biala, 43309 Bielsko-Biala, Poland; 32 Institute of Technology, The State University of Applied Sciences, 82300 Elblag, Poland; 33 Department of Marine Biotechnology, University of Gdansk, 81378 Gdynia, Poland; 34 Department of Animal Biology, Plant Biology and Ecology, University of Jaen, 23701 Jaen, Spain; 35 Institute of Nature Conservation, Polish Academy of Sciences, 31-120 Krakow, Poland; 36 European Regional Centre for Ecohydrology of the Polish Academy of Sciences, 90364 Lodz, Poland; 37 Department of Hydrobiology and Protection of Ecosystems, University of Life Sciences in Lublin, 20262 Lublin, Poland; 38 Department of Icthyology, Hydrobiology and Aquatic Ecology, Inland Fisheries Institute, 10719 Olsztyn, Poland; 39 Department of Water Protection Engineering, University of Warmia and Mazury, 10-720 Olsztyn, Poland; 40 Department of Tourism, Recreation and Ecology, University of Warmia and Mazury, 10-720 Olsztyn, Poland; 41 Department of Water Protection, Adam Mickiewicz University, 61614 Poznan, Poland; 42 Department of Hydrobiology, Adam Mickiewicz University, 61614 Poznan, Poland; 43 Institute of Environmental Engineering, Poznan University of Technology, 60965 Poznan, Poland; 44 Faculty of Biology, University of Warsaw, 02-096 Warsaw, Poland; 45 Department of Environmental Improvement, Faculty of Civil and Environmental Engineering, Warsaw University of Life Sciences—SGGW, 02-787 Warsaw, Poland; 46 Department of Hydraulic Engineering, Faculty of Civil and Environmental Engineering, Warsaw University of Life Sciences—SGGW, 02-787 Warsaw, Poland; 47 Department of Freshwater Protection, Institute of Environmental Protection- National Research Institute, 01-692 Warsaw, Poland; 48 Department of Plant Ecology and Environmental Conservation, Faculty of Biology, University of Warsaw, 02-089 Warsaw, Poland; 49 Centro de Investigação da Montanha, Instituto Politécnico de Bragança, Campus de Santa Apolónia 5300-253 Bragança, Portugal; 50 Instituto Dom Luiz, University of Lisbon, 1749016 Lisbon, Portugal; 51 Institute of Earth Sciences Jaume Almera, ICTJA, CSIC, 08028 Barcelona, Spain; 52 Interdisciplinary Centre of Marine and Environmental Research (CIIMAR) and University of Porto, 4450-208 Matosinhos, Portugal; 53 Research Center in Biodiversity and Genetic Resources (CIBIO-Azores), InBIO Associated Laboratory, Faculty of Sciences and Technology, University of the Azores, 9501-801 Ponta Delgada, Portugal; 54 Faculty of Natural Sciences and Mathematics, SS Cyril and Methodius University, 1000 Skopje, Macedonia; 55 National Reference Center for Hydrobiology, Public Health Authority of the Slovak Republic, 82645 Bratislava, Slovakia; 56 Department of Water Quality, Slovenian Environmental Agency, 1000 Ljubljana, Slovenia; 57 Department of Genetic Toxicology and Cancer Biology, National Institute of Biology, 1000 Ljubljana, Slovenia; 58 Department of Civil Engineering, University of A Coruña, 15192 A Coruña, Spain; 59 Department of Evolutionary Biology, Ecology, and Environmental Sciences, University of Barcelona, 08028 Barcelona, Spain; 60 Department of Limnology and Water Quality, AECOM U.R.S, 08036 Barcelona, Spain; 61 Department of Biology, University of Cádiz, 11510 Puerto Real, Cádiz, Spain; 62 Catalan Institute for Water Research (ICRA), 17003 Girona, Spain; 63 Department of Ecology, University of Granada, 18071 Granada, Spain; 64 R&D Department Environmental Engineering, 3edata, 27004 Lugo, Spain; 65 Department of Ecology, University of Malaga, 29071 Malaga, Spain; 66 Centro de Investigacións Cientificas Avanzadas (CICA), Facultade de Ciencias, Universidade da Coruña, 15071 A Coruña, Spain; 67 Cavanilles Institute of Biodiversity and Evolutionary Biology, University of Valencia, 46980 Paterna Valencia, Spain; 68 Department of Microbiology and Ecology, University of Valencia, 46100 Burjassot, Spain; 69 Department of Biology, Lund University, 22362 Lund, Sweden; 70 Department of Forest Engineering, University of Cankiri Karatekin, 18200 Cankiri, Turkey; 71 Department of Ecology and Genetics, Limnology, Uppsala University, 75236 Uppsala, Sweden; 72 Department of Ecology and Genetics, Erken Laboratory, Uppsala University, 76173 Norrtalje, Sweden; 73 Department of Aquatic Ecology, Netherlands Institute of Ecology (NIOO-KNAW), 6700 Wageningen, The Netherlands; 74 Department of Environmental Sciences, Wageningen University & Research, 6700 Wageningen, The Netherlands; 75 Department of Environmental Sciences, Aquatic ecology and water quality management group, Wageningen University, 6700 Wageningen, The Netherlands; 76 Society for the Protection of Prespa, 53077 Agios Germanos, Greece; 77 Institute for Water and Wetland Research, Department of Aquatic Ecology and Environmental Biology, Radboud University Nijmegen, 6525 AJ Nijmegen, The Netherlands; 78 Research Institute RIKILT, BU Contaminants & Toxins, Wageningen University, 6708 WB Wageningen, The Netherlands; 79 Laboratoire Microorganismes - Génome et Environnement, Université Clermont Auverge, Campus Universitaire des Cézeaux, Aubiere Cedex 63178, France; 80 Department of Biological Sciences, Virginia Tech, 24061 Virginia, USA; 81 Institute of Marine Sciences, University of North Carolina at Chapel Hill, 28557 North Carolina, USA; 82 National Institute of Environmental Health, 1097 Budapest, Hungary; 83 Department of Molecular Biology and Genetics, Gaziosmanpasa University, 60250 Merkez, Turkey; 84 Department of Fisheries and Aquaculture, Ankara University, 6100 Ankara, Turkey; 85 Department of biology, Middle East Technical University, 6800 Ankara, Turkey; 86 Department of Biology, Balikesir University, 10145 Balikesir, Turkey; 87 Institute of Marine Sciences, Department of Oceanography, Middle East Technical University, 06800 Ankara, Turkey; 88 Department of Environmental Engineering, Abant Izzet Baysal University, 14280 Bolu, Turkey; 89 Department of Environment and Resource Management, Mediterranean Fisheries Research Production and Training Institute, 7090 Antalya, Turkey; 90 Department of Bioengineering, Bursa Technical University, 16310 Bursa, Turkey; 91 Department of Biology, Hitit University, 19040 Corum, Turkey; 92 Department of Basic Science, Ataturk University, 25240 Erzurum, Turkey; 93 Department of Biology, Giresun University, 28100 Giresun, Turkey; 94 Republic of Turkey Ministry of Food Agriculture, Fisheries Research Institute, 32500, Eğirdir, Isparta, Turkey; 95 Department of Freshwater Resource and Management, Faculty of Aquatic Sciences, Istanbul University, 34134 Istanbul, Turkey; 96 Faculty of Aquaculture, Mersin University, 33160 Mersin, Turkey; 97 Institute of Marine Sciences, Marine Biology and Fisheries, Middle East Technical University, 33340 Mersin, Turkey; 98 Department of Biology, Sakarya University, 54187 Sakarya, Turkey; 99 Department of Biological and Environmental Sciences, University of Stirling, FK9 4LA Stirling, UK; 100 School of Pharmacy and Life Sciences, Robert Gordon University, AB10 7GJ Aberdeen, UK; 101 Research department for Limnology, University of Innsbruck, 5310 Mondsee, Austria; 102 Independent graphic designer and illustration, 6a Everton Ave, Cabra, D07 PN2W Dublin, Ireland; 103 Independent graphic designer, 11 Aravon Ct, A98 D529 Bray, Ireland; 104 Agri-Food & Biosciences Institute, 18A Newforge Lane, BT9 5PX Belfast, UK.

**Keywords:** Limnology, Water resources, Climate-change ecology

## Abstract

Under ongoing climate change and increasing anthropogenic activity, which continuously challenge ecosystem resilience, an in-depth understanding of ecological processes is urgently needed. Lakes, as providers of numerous ecosystem services, face multiple stressors that threaten their functioning. Harmful cyanobacterial blooms are a persistent problem resulting from nutrient pollution and climate-change induced stressors, like poor transparency, increased water temperature and enhanced stratification. Consistency in data collection and analysis methods is necessary to achieve fully comparable datasets and for statistical validity, avoiding issues linked to disparate data sources. The European Multi Lake Survey (EMLS) in summer 2015 was an initiative among scientists from 27 countries to collect and analyse lake physical, chemical and biological variables in a fully standardized manner. This database includes *in-situ* lake variables along with nutrient, pigment and cyanotoxin data of 369 lakes in Europe, which were centrally analysed in dedicated laboratories. Publishing the EMLS methods and dataset might inspire similar initiatives to study across large geographic areas that will contribute to better understanding lake responses in a changing environment.

## Background & Summary

Eutrophication still is the primary process threatening lakes and reservoirs and the services they provide, like good quality drinking water, irrigation, fisheries and recreational opportunities. Anthropogenic eutrophication is responsible for massive algal blooms. Cyanobacteria have diverse functional traits that allow them to proliferate under various environmental conditions^1^. The frequency and size of cyanobacterial blooms is increasing globally^2,3^. Excessive cyanobacterial biomass reduces light penetration and enhances anoxia in the hypolimnion, thus reducing species habitats and biodiversity^4^. Moreover, the toxins produced by bloom-forming cyanobacteria present a considerable risk to drinking water^5^ and pose a substantial economic cost^6,7^.

Climate change, through direct and indirect effects of warming, increasingly plays a role in changing physico-chemical and biological properties of aquatic ecosystems^8–11^, contributing to the global increase in cyanobacterial blooms. Although optimal growth temperatures vary widely between cyanobacterial strains and species, as well as for their eukaryotic competitors, their growth rate increases faster with temperature than for other phytoplankton groups^9,12^, Longer ice-free seasons, reduced winter overturn and enhanced water column stability in summer may all indirectly favour cyanobacterial blooms. In deep peri-alpine lakes for instance, water column stability has increased by 20% in a response to warming of the atmosphere^13^.

Interactions between nutrients and temperature-related changes are expected^14–16^. However, it is still uncertain to what extent and following which mechanisms nutrients and temperature will interact to amplify blooms. Climate forcing of blooms will differ among regions^17^. For example, at high latitudes and equatorial areas, intense precipitation events are expected (IPCC7) to increase nutrient enrichment of water bodies from enhanced surface runoff and groundwater discharge^4^, whereas at low and mid-latitude continental interiors, droughts^18^ may reduce river flow rates, increase lake residence times and thereby promote cyanobacteria. In hyper-eutrophic systems, high algal biomass may even increase local temperature due to enhanced light absorption^19^. Thus, high nutrient concentrations may promote warmer temperatures, giving a competitive advantage to buoyant cyanobacteria over non-buoyant algal species^4^. All of these interactions between nutrients and temperature may vary with lake depth. Shallow lakes respond more directly to nutrients and temperature than deep lakes, which, in contrast, are more typically subjected to the indirect effects of the aforementioned drivers^20^.

Hence, a complex interplay of regional (climate), local (nutrients, lake morphometry) and biological (species, functional groups) lake variables determines cyanobacterial bloom formation. Consequently, studies covering different regions and lake types are needed to disentangle the relative importance of the environmental predictors and their interactions. The European Multi Lake Survey (EMLS) obtained a deeper insight into cyanobacterial dynamics under different ecosystem variables across Europe. A space-for-time substitution, where contemporary spatial phenomena are studied in many lakes, instead of long-term temporal studies in a limited number of lakes^21^, was used. The survey took place in summer 2015, Europe’s third hottest summer on record, and comprised scientists from 27 countries that sampled 369 lakes only once. In this way, environmental gradients across wide geographic scales were covered with relatively little effort and with higher cost efficiency. We sampled in summer as cyanobacterial blooms are a distinct feature of summer phytoplankton^22^, during the locally warmest period, in order to test for temperature effects on cyanobacteria. In EMLS, standardized sampling procedures were strictly followed to ensure data homogeneity and eliminate site or operator related observation effects. Finally, lake samples for nutrients, algal pigments and toxins were analysed in dedicated laboratories by one person on one machine, minimizing variation in analytical errors.

Apart from providing a solid research dataset on which several analyses are being conducted, the EMLS helped to enhance the standards for limnological data collection and stimulate international collaboration. A subset of the EMLS toxin dataset has already been used in a recent publication to show how the distribution in cyanobacterial toxins and toxin quota was determined by both direct and indirect effects of temperature^23^. Here we additionally present the data for all lakes, including those without toxins for the full set of data. The intention is that this publication of the EMLS dataset will further demonstrate the feasibility and value of snapshot surveys, and encourage similar programs in a continuously changing environment, i.e. at times when datasets covering large geographical gradients are in great demand.

## Methods

### Organization

To make EMLS a robust survey we bridged two European COST Actions^24^, CyanoCOST^25,26^ (Cyanobacterial blooms and toxins in water resources: occurrence impacts and management) and NETLAKE^27^ (Networking Lake Observatories in Europe), planting the idea and promoting the benefits of an extended collaboration amongst researchers from all over Europe. This research was expanded to many other scientists not directly involved in these COST Actions.

The EMLS protocols required that each group of data providers collected and handled the samples following the same standards. Therefore, the steps outlined below had to be followed by all participant groups to ensure standardized sampling, sample processing and analyses, resulting in a homogenous dataset. Any deviations from these protocols were recorded in the metadata spreadsheets, and these data were handled with care after contacting the data collectors. To ensure that protocols were fit-for-purpose and understandable to everyone, we invited representatives of each country involved in the CyanoCOST and NETLAKE actions to a three-day training workshop in Evian-Les-Bains, France. During the workshop, participants discussed all aspects of EMLS and considered limitations in the financial and logistic means for given countries, without compromising research quality. They finalized the protocols and obtained hands-on experience in using them. To increase the number of studied lakes and achieve adequate spatial variation, representatives of each country acted as EMLS-ambassadors, reaching-out to further collaborators within their own countries and disseminating the decisions and protocols of the EMLS.

**Figure 1:**
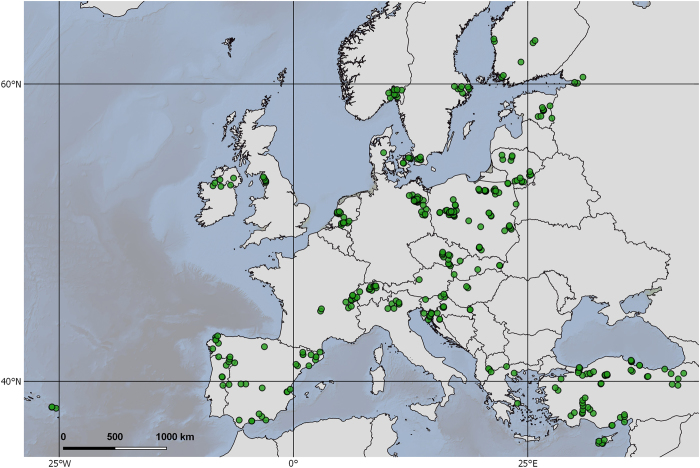
Map showing the locations of the 369 lakes sampled during the European Multi Lake Survey in summer 2015.

The EMLS was a collective effort, which means that each participating group used their own financial means to conduct their sampling as well as provided the personnel and facilities needed. Since the EMLS was a zero-funding effort, individual countries mainly contributed with samples from lakes that they routinely sample anyway, especially lakes with a history of eutrophication, given the implications for lake management. Although this results in a bias towards productive lakes, unfortunately it also reflects reality, with many lakes in Europe still suffering from eutrophication. A total of 369 lakes were sampled, spanning from Cyprus to Finland, and from the Asian part of Turkey to the Portuguese Azores islands (Fig. 1).

**Figure 2:**
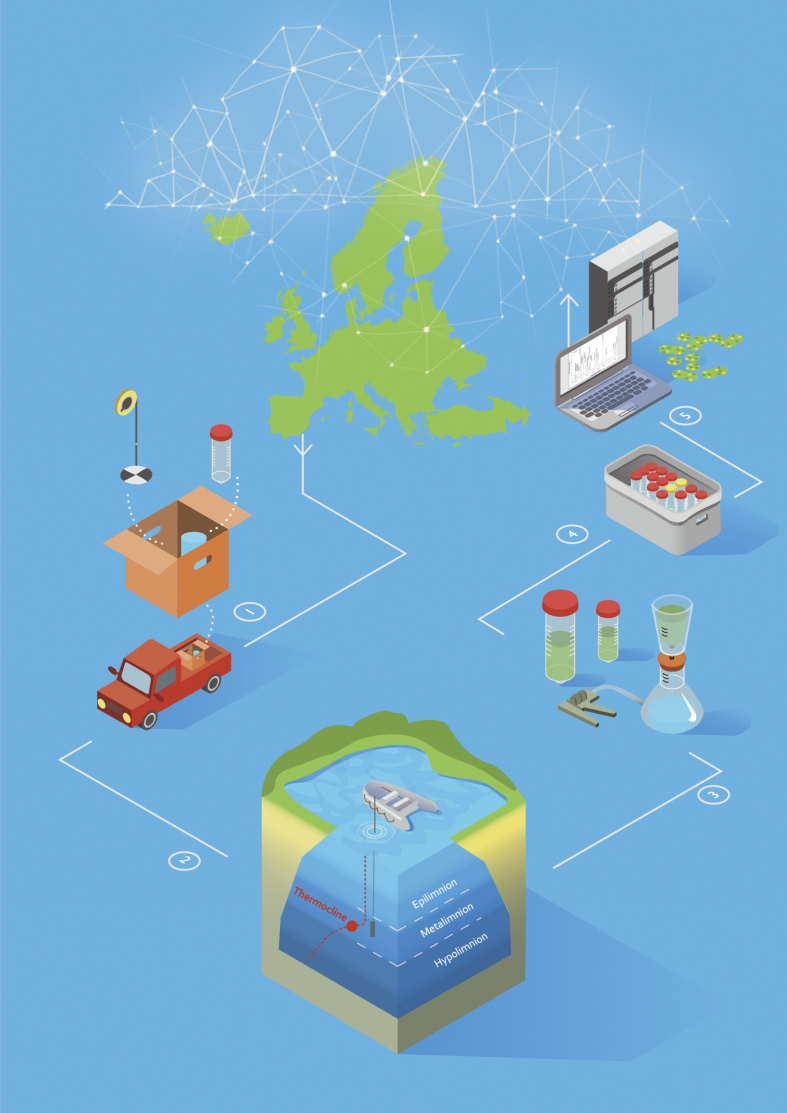
Schematic overview of the European Multi Lake Survey (EMLS). CyanoCOST and NETLAKE members performed the sampling routine by: 1. Preparing the sampling material to meet the standardized procedures 2. Accessing the lake site and acquiring integrated water samples from the bottom of the thermocline up to the water surface 3. Processing and preserving the water samples based on requirements for subsequent analysis 4. Shipping samples to a central receiving laboratory 5. Analysing nutrient, pigment and toxin concentrations in dedicated laboratories. After quality control checks and data integration the dataset returns to the European network and becomes publically available for further research. Created by Sarah O’Leary and Eilish Beirne.

The workflow for organizing the EMLS consortium is presented as an infographic (Fig. 2). It illustrates the logistics from organizing the local surveys (1) through obtaining the samples (2), to processing and shipping them to the analytical laboratories (3-5). The data obtained from the field as well as laboratory-analyses was quality controlled and integrated into a unique dataset before making available to the EMLS network and to the rest of the scientific community. The methods of data acquisition and laboratory analyses that are described below are expanded versions of descriptions in our related work^23^.

### Data acquisition

#### Date and location (*in situ*)

The field methods for the EMLS were designed to be completed within one field day for each lake, but typically the sampling itself could be completed within two hours. Remote, poorly accessible lakes required more time to reach and field crews had to plan accordingly. To optimize time investment, lakes in close proximity were covered in one sampling trip. Each sampling group was responsible to organize and prepare the sampling material and equipment for their sampling campaign. In several cases, sampling groups of different areas or even countries collaborated and shared material such as instruments and boats.

Each EMLS data collector team had to identify the right sampling period, defined as the warmest two-week period in summer, based on long-term air temperature data in each region, covering the last 10 or more years. This predefined time-period served to minimize confounding effects of seasonality. Each lake was sampled within this time window (Date; Table 1).

The sampling location for each lake was defined as the central point of the lake. If a particular lake had been previously sampled at a specific location for long-term monitoring, the sample location from the long-term monitoring was used instead of the lake centre. For lakes with more than one relatively isolated basin, individual basins were sampled separately when possible, and indicated as such in the dataset (e.g. TR_BEY_I and TR_BEY_II). The latitude and longitude of the sampling location were recorded with a GPS device and provided in decimal degrees according to the WGS84 coordinate system.

If cyanobacterial surface blooms (defined as the presence of a visible surface scum) were present close to where the team entered the lake or in close proximity to the sampling point, a second sampling location was considered. A scoop surface sample, using a small sealable container like a Falcon tube, of the cyanobacterial scum was acquired along with the water column sample. The location of the scum sample was noted. Data collectors also provided - when available – maximum depth, mean depth and altitude (Table 1) of the lake.

#### Temperature and Secchi depth (*in situ*)

Temperature profiles were measured with available probes, such as various CTDs or a Fluoroprobe (BBE Moldaenke). If no profiling instrument was available, water samples were taken at 0.5 m intervals from the lake surface to the bottom of the thermocline (top of the hypolimnion) and water temperature was measured using hand-held thermometers directly after sampling. Here, the thermocline range was defined as the depth interval at which the rate of temperature decreased at least 1 °C per metre. The bottom of the thermocline (where temperature no longer decreased by 1 °C per meter), defined the sampling depth in the case of stratified lakes. For lakes where a thermocline was not observed (mostly shallow lakes), the temperature profile was measured until the lake bottom. In this case, the sampling depth was determined at 0.5 m above the lake bottom. From the temperature profiles, we obtained surface and epilimnetic temperature (Table 1) as the average temperature from surface until the bottom of the thermocline. The temperature profiles were also used during data analysis, to calculate the location where the thermocline lies even between two temperature measurement depths, which corresponds to where the thermocline is the most stable (point of maximum buoyancy frequency). To calculate this thermocline depth (ThermoclineDepth_m, Table 1) we used the command thermo.depth from the R package rLakeAnalyzer^28^. The Secchi depth (Table 1) was also recorded using a Secchi disk to the nearest 0.05 m.

#### Integrated water sample (*in situ*)

All data collectors constructed a simple device, known as the “Anaconda”, using a stoppered hose of the correct length in order to acquire the epilimnetic sample. The hosepipe was lowered with the bottom end open into the water column until the right depth (see above). When the hosepipe was vertical and the water level was visible at the surface layer of the hosepipe then the stopper was inserted to create hydrostatic pressure. The bottom end of the hosepipe was pulled-up with a rope to the surface to collect the sample in a bucket. The diameter of the hosepipe was appropriate to sample the required water volume (about 5–10 L for hypertrophic and eutrophic, 15–30 L for mesotrophic and oligotrophic lakes) for the analyses, in an acceptable number of runs. The first three sampling runs served the purpose of rinsing the hosepipe, the sampling bucket and the plastic rod. The subsequent runs were the water sample taken for analysis. The water sample in the bucket was mixed adequately before being divided into different bottles for further processing prior to analysis.

All samples were shipped frozen using dry ice in Styrofoam boxes. Shipping and storage of the EMLS samples was centralized at the University of Wageningen (The Netherlands). There, samples were sorted and sent to the dedicated laboratories for further analysis. Each of the nutrients, pigments and toxins analyses were done in one dedicated laboratory, by one operator on one machine, to minimize analytical errors and maximize integration of the datasets. Specifically, the nutrients, microcystins and nodularin analyses were done at the University of Wageningen, the pigment analysis at the University of Amsterdam and the cylindrospermopsin and anatoxin analysis at the German Environment Agency.

#### Total and Dissolved Nutrients (laboratory)

For analyses of total phosphorus (TP_mgL, Table 1) and nitrogen (TN_mgL, Table 1), unfiltered water subsamples of 50 mL were stored in -20 °C until shipping. For dissolved nutrients: orthophosphate (PO4_ugL, Table 1), nitrite/nitrate (NO3NO2_mgL, Table 1) and ammonium (NH4_mgL, Table 1), a volume of 250 mL was filtered through 47mm Glass fibre filters (GF/C or GF/F or similar), the filtrate was sampled in a PE bottle and stored at −20 °C. Before the collection of the nutrient samples, all polyethylene collection bottles with their screw caps were acid washed overnight in 1M HCl and rinsed with demineralized water and lake water before collection. Nutrients were measured according to Dutch NEN standards, using a Skalar SAN+ segmented flow analyser (Skalar Analytical BV, Breda, NL) with UV/persulfate digestion integrated in the system. The total phosphorus and orthophosphate were analysed conforming NEN^29^, the ammonium and total nitrogen according to NEN^30^ and the nitrite/nitrate following NEN^31^. The limit of detection was 0.02 mg/L for total phosphorus and ammonium, 0.2 mg/L for total nitrogen, 0.01 mg/L for nitrite/nitrate and 0.004 mg/L for orthophosphate.

#### Pigment analysis (laboratory)

For pigment analysis, a volume of 50–250 mL for hypertrophic and eutrophic lakes and 500–1000 mL for mesotrophic to oligotrophic lakes was filtered through 47mm glass fibre filters (GF/C or GF/F or similar) using a filtration device. Filters were stored at −20 °C in the dark until shipping. The analysis of pigments was modified from the method described by Van der Staay *et al.*^32^. All filters were freeze dried for 6 h. Filters were cut in half, placed in separate Eppendorf tubes, and kept on ice until the end of the extraction procedure. In each tube, 600 μl of 90% acetone were added with a small amount of 0.5 mm glass beads. To extract the pigments from the phytoplankton cells, filters were placed on a bead-beater for one minute. To increase the extraction yields, samples were placed in an ultrasonic bath for ten minutes. This procedure was repeated twice to ensure a complete extraction of the total pigment content of the filters. To achieve binding of the pigments during the High-Performance Liquid Chromatography (HPLC) analysis, 300 μl of a Tributyl Ammonium Acetate (1.5%) and Ammonium Acetate (7.7%) mix were added to each tube. Lastly, samples were centrifuged at 15, 000 rpm at 4 °C for ten minutes. 35 μl of the supernatant from both Eppendorf tubes of a filter were transferred into an HPLC glass vials. Pigments were separated on a Thermo Scientific ODS Hypersil column (250 mm × 3 mm, particle size 5 μm) in a Shimadzu HPLC machine and using a KONTRON SPD-M2OA diode array detector. We identified 12 different pigments (chlorophyll-a, chlorophyll-b, zeaxanthin, diadinoxanthin, fucoxanthin, diatoxanthin, alloxanthin, peridinin, chlorophyll-c2, echinenone, lutein and violaxanthin, Table 1) based on their retention time and absorption spectrum and quantified by means of pigment standards. The limit of detection (LOD) and for a 250 mL sample was: 0.094 μg/L for chlorophyll-a, 0.061 μg/L for chlorophyll-b, 0.034 μg/L for zeaxanthin, 0.053 μg/L for diadinoxanthin, 0.067 μg/L for fucoxanthin, 0.029 μg/L for diatoxanthin, 0.023 μg/L for alloxanthin, 0.085 μg/L for peridinin, 0.028 μg/L for chlorophyll-c2, 0.031 μg/L for echinenone, 0.027 μg/L for lutein and 0.075 μg/L for violaxanthin. The limit of quantification (LOQ) and for a 250 mL sample was: 0.315 μg/L for chlorophyll-a, 0.202 μg/L for chlorophyll-b, 0.115 μg/L for zeaxanthin, 0.177 μg/L for diadinoxanthin, 0.224 μg/L for fucoxanthin, 0.098 μg/L for diatoxanthin, 0.077 μg/L for alloxanthin, 0.284 μg/L for peridinin, 0.093 μg/L for chlorophyll-c2, 0.104 μg/L for echinenone, 0.089 μg/L for lutein and 0.250 μg/L for violaxanthin. In the cases where no pigment signal was detected, the respective pigment was considered absent and noted as 0 μg/L in the dataset. If the calculated pigment concentration in the dataset is above the limit of detection (qualitatively detected signal) but below the quantification limit (too small to quantify), we suggest the assignment of a very small value of half the detection limit to enable the inclusion of these samples in statistical analyses (if applicable). Alternatively, other statistical approaches that account for data censoring can be followed based on the research question and the statistical analysis followed (for suggestions see^33^).

#### Cyanotoxin analysis (laboratory)

For toxin analyses, a volume of 50–250 mL for hypertrophic and eutrophic lakes and 500-1000 mL for mesotrophic to oligotrophic lakes, was filtered through 47 mm Glass fibre filters (GF/C or GF/F or similar) using a filtration device. Filters were stored at −20 °C until shipping. In the laboratory, frozen filters were transferred to 8 mL glass tubes and freeze-dried (Alpha 1-2 LD, Martin Christ Gefriertrocknungsanlagen GmbH, Osterode am Harz, Germany). The freeze-dried filters were used for the Liquid Chromatography with tandem Mass Spectrometry detection (LC-MS/MS) analysis of microcystins, nodularin, cylindrospermopsin and anatoxin as described below. In the cases where no toxin signal was detected, the respective toxin was considered absent and noted as 0 μg/L in the dataset. A similar approach as in the section “pigment analysis” can be followed for toxin concentrations in the dataset that fall above the detection limit but below the quantification limit, as we did in^23^.

#### Microcystins and nodularin analysis (laboratory)

For the extraction of microcystins and nodularin, 2.5 mL of 75% hot methanol – 25% ultrapure water (v/v) was added to the freeze-dried filters, which were then sealed with a screw cap and placed for half an hour at 60 °C. Subsequently, the extract was transferred to a clean 8 mL glass tube. This extraction procedure was performed three times for each filter. The supernatants of the repeated extraction procedure were combined to a final volume of 7.5 mL and then dried in a Speedvac (Thermo Scientific Savant SPD121P, Asheville, NC, USA). After that, the extracts were reconstituted in 900 μL 100% MeOH. The reconstituted samples were transferred into 2 mL Eppendorf vials with a 0.22 μm cellulose-acetate filter and centrifuged for 5 min at 16, 000×g (VWR Galaxy 16DH, Boxmeer, Netherlands). Filtrates were transferred to amber glass vials for the analysis.

The LC-MS/MS analysis was performed on an Agilent 1200 LC and an Agilent 6410A QQQ (Waldbronn, Germany). The extracts were separated using a 5 μm Agilent Eclipse XDB-C18 (4.6 mm, 150 mm column, Agilent Technologies, Waldbronn, Germany) at 40 °C. The mobile phase consisted of Millipore water (v/v, eluent A) and acetonitrile (v/v, eluent B) both containing 0.1% formic acid at a flow rate of 0.5 mL/min with the following gradient program: 0–2 min 30% B, 6–12 min 90% B, with a linear increase of B between 2 and 6 min and a 5 min post run at 30% B (as described in^32^). The injection volume was 10 μl. Identification of the eight MC variants (MC_dmRR, MC_RR, MC_YR, MC_dmLR, MC_LR, MC_LY, MC_LW and MC_LF, Table 1) and nodularin (NOD) was performed in the positive Multiple Reaction Monitoring (MRM) with the following transitions: MC_dmRR 512.8 m/z [M + H]^+^ to 135.1 quantifier, MC_RR 519.8 m/z [M + H]^+^ to 135.1 quantifier, MC_YR 523.3 m/z [M + H]^+^ to 135.1 quantifier, MC_dmLR 491.3 m/z [M + H]^+^ to 847.6 quantifier, MC_LR 498.3 m/z [M + H]^+^ to 135.1 quantifier, MC_LY 868.4 m/z [M + H]^+^ to 163.0 quantifier, MC_LW 891.5 m/z [M + H]^+^ to 163.0 quantifier, MC_LF 852.5 m/z [M + H]^+^ to 163.0 quantifier and NOD 825.5 m/z [M + H]^+^ to 135.1 quantifier^34^. Mass spectrometric settings are given in^35^. Each MC variant was quantified against a calibration curve. The calibration curves were made using certified calibration standards obtained from DHI LAB Products (Hørsholm, Denmark). The limit of detection (LOD) and quantification (LOQ) for a 250 mL sample was: 0.0489 μg/L for MC_dmRR, 0.0358 μg/L for MC_RR, 0.0050 μg/L for MC_YR, 0.0054 μg/L for MC_dmLR, 0.0086 μg/L for MC_LR, 0.0817 μg/L for MC_LY, 0.0531 μg/L for MC_LW, 0.0206 μg/L for MC_LF and 0.0048 μg/L for NOD.

#### Cylindrospermopsin and anatoxin analysis (laboratory)

For the extraction of cylindrospermopsin (CYN, Table 1) and anatoxin-a (ATX, Table 1), 1.5 mL of 0.1% formic acid was added to the freeze-dried filters. Filters were sonicated for 10 min, shaken for 1 hour and then, centrifuged. This extraction procedure was repeated two more times and the combined supernatants were dried in a Speedvac (Eppendorf, Germany). Prior to analysis the dried extracts were re-dissolved in 1 mL 0.1% formic acid and filtered (0.2 μm, PVDF, Whatman, Maidstone, UK).

LC-MS/MS analysis was carried out on an Agilent 2900 series HPLC system (Agilent Technologies, Waldbronn, Germany) coupled to a API 5500 QTrap mass spectrometer (AB Sciex, Framingham, MA, USA) equipped with a turbo-ion spray interface. The extracts were separated using a 5 mm Atlantis C18 (2.1 mm, 150 mm column, Waters, Eschborn, Germany) at 30 °C. The mobile phase consisted of water (v/v, eluent A) and methanol (v/v, eluent A) both containing 0.1% formic acid, and was delivered as a linear gradient from 1% to 25% B within 5 min at a flow rate of 0.25 mL/min. The injection volume was 10 μL. Identification of CYN and ATX was performed in the positive MRM mode with the following transitions: CYN m/z 416.1 [M + H]^+^ to 194 (quantifier) and 416.1/176, and ATX m/z 166.1 [M + H]^+^ to 149, 166.1/131, 166.1/91, 166.1/43 (quantifier). Mass spectrometric settings are given in^36^. Certified reference standards were purchased from National Research Council (Ottawa, ON, Canada). The limit of detection (LOD) for both ATX and CYN was 0.0001 μg/L and the limit of quantification (LOQ) was 0.0004 μg/L for a 250 mL sample.

#### Code availability

Custom-made codes in R 3.3.3^37^. were used to combine the datasets, trace missing data and inconsistencies. The codes are available in Zenodo: https://zenodo.org/record/1219878#.Wtcc4S5ubRZ

The GeoNode open source platform version 2.0 has been used for sharing the EMLS datasets among the partners. GeoNode is a web-based application that facilitates the visualization, download, sharing, and collaborative use of geospatial data through web services. GeoNode can be easily obtained at http://geonode.org/ as it is freely available under a GNU General Public License.

QGIS 2.18 Las Palmas was used for creating, managing and uploading the ESRI shapefile layers into the GeoNode platform.

## Data Records

The final dataset includes all lake, environmental, nutrient, pigment and toxin data in one data table. The description of each feature in the table can be found in Table 1. The data table is made freely available as a static copy, through direct download from the online Environmental Database Initiative (EDI) and it is provided under the name “EMLSdata_10Aug_afterRev_dateformated.csv” (Data Citation 1).

The data table is also available at the GeoNode platform (http://gleon.grid.unep.ch/), where it can be downloaded through the provided web services that secure accessibility to data by using interoperable standards as provided by the Open Geospatial Consortium (OGC). The OGC- compliant web services available are 1. the Web Map Service (WMS 1.1.1) and 2. the Web Map Tile Service (WMTS 1.0.0) for accessing the maps; 3. the Web Feature Service (WFS 1.1.0) for accessing vector data and 4. Catalogue Service for the Web (CSW 2.0.2) to access the metadata. These interoperable web service endpoints enable the user to easily access and/or integrate these datasets in their desktop, web-based client, or own workflows.

The user can find and download several features of the data table as vector layers under the tab “Layers”. In the interactive tab “Maps”, the user can visualise and download datasets of combined “Layers”, or create their own maps using the available layers. The vector layers are provided in several formats such as: ESRI shapefile, Geography Markup Language (GML) and Keyhole Markup Language (KML), JPEG, pdf etc.

Both the database and the GeoNode platform can be easily updated with the expected data from DNA and flowcytometry analysis (to follow). As new surveys may be organized following the protocols of this paper, this data can also be easily included in the database and GeoNode platform.

## Technical Validation

All data received from field observers (i.e. data in the tables: Lake Data & Metadata, Sampled data and depth profiles) were checked by a data curator before uploaded into the database.

### Tracing missing data

Participating data collectors provided a field datasheet and a metadata sheet for each lake. All sheets for each sampling event and lake metadata which did not have matching records were double checked, and errors were corrected when found. A custom-made code generated proofing reports for each table, highlighting which lakes or basins had missing data.

### Location data

All latitude and longitude records for each lake were verified by checking visually the provided locations on google maps. Lakes were marked as verified under the following conditions: (1) the location on google maps, using either satellite or map view, was found for a lake which matched the provided name, (2) the location on google maps was located close to a water body (approximately 1 km or closer) with a matching name, (3) the location was in or beside a lake which had no name or a different name, but the name provided matched with names of regions in the area (i.e. this represents cases where lakes were named according to their closest city, or region), or (4) the provided location was near an unnamed lake in the correct country where there were no other lakes nearby. All other cases were considered unverified and the data collectors were contacted to provide the right location (Latitude and longitude in decimal degrees; WGS84).

### Laboratory Analyses

The nutrient, pigment and toxin concentrations were analysed centrally by certified laboratories that have optimized those specific analytical methods. Given that, these data are assumed to be correct.

## Additional information

**How to cite this article**: Mantzouki, E. *et al.* A European Multi Lake Survey dataset of environmental variables, phytoplankton pigments and cyanotoxins. *Sci. Data*. 5:180226 doi: 10.1038/sdata.2018.226 (2018).

**Publisher’s note**: Springer Nature remains neutral with regard to jurisdictional claims in published maps and institutional affiliations.

## Supplementary Material



## Figures and Tables

**Table 1:** Column names, column description and explanation/format or unit for each feature of the lake file.

Column name	Description	Explanation or format or unit
**Lake_ID**	A code given to each studied lake	e.g. TR_BEY_I,
		TR = country code (Turkey),
		BEY = three first letters of lake name (Lake Beysehir),
		I = if more than one lake basins were sampled
**Date**	Date of sample collection	YYYY-MM-DD
**LakeName**	Name of lake that is commonly used	Name
**LabName**	The name of the laboratory that organized the local sampling	Name
**Country**	Geographic location of lake	Name
**Latitude**	Latitude coordinate of lake	Decimal degrees (WGS84)
**Longitude**	Longitude coordinate of lake	Decimal degrees (WGS84)
**Altitude_m**	Elevation of lake surface relative to sea level	Metres
**MaximumDepth_m**	Maximum depth of lake	Metres
**MeanDepth_m**	Mean depth of lake	Metres
**SecchiDepth_m**	Secchi depth reading	Metres
**SamplingDepth_m**	Depth of sample collection	Metres
	(lower limit of the sampling interval)	
**ThermoclineDepth_m**	Location of the thermocline	Metres
		NA signifies a non-stratified lake (no thermocline detected)
**SurfaceTemperature_C**	Lake water temperature at 0.5 m depth	Degrees Celsius
**EpilimneticTemperature_C**	Averaged lake water temperature from surface until bottom of the thermocline	Degrees Celsius
**TP_mgL**	Concentration of total phosphorus	Milligrams per litre
**TN_mgL**	Concentration of total nitrogen	Milligrams per litre
**NO3NO2_mgL**	Concentration of nitrates and nitrites (dissolved nutrients)	Milligrams per litre
**NH4_mgL**	Concentration of ammonia (dissolved nutrients)	Milligrams per litre
**PO4_ugL**	Concentration of ortho-phosphate (dissolved nutrients)	Micrograms per litre
**Chlorophylla_ugL**	Concentration of photosynthetic pigment chlorophyll-a	Micrograms per litre
**Chlorophyllb_ugL**	Concentration of photosynthetic pigment chlorophyll-b	Micrograms per litre
**Zeaxanthin_ugL**	Concentration of photosynthetic pigment zeaxanthin	Micrograms per litre
**Diadinoxanthin_ugL**	Concentration of photosynthetic pigment diadinoxanthin	Micrograms per litre
**Fucoxanthin_ugL**	Concentration of photosynthetic pigment fucoxanthin	Micrograms per litre
**Diatoxanthin_ugL**	Concentration of photosynthetic pigment diatoxanthin	Micrograms per litre
**Alloxanthin_ugL**	Concentration of photosynthetic pigment alloxanthin	Micrograms per litre
**Peridinin_ugL**	Concentration of photosynthetic pigment peridinin	Micrograms per litre
**Chlorophyllc2_ugL**	Concentration of photosynthetic pigment chlorophyll-c2	Micrograms per litre
**Echinenone_ugL**	Concentration of photosynthetic pigment echinenone	Micrograms per litre
**Lutein_ugL**	Concentration of photosynthetic pigment lutein	Micrograms per litre
**Violaxanthin_ugL**	Concentration of photosynthetic pigment violaxanthin	Micrograms per litre
**MC_YR.ugL**	Concentration of cyanobacterial hepatotoxin microcystin YR	Micrograms per litre
**MC_dmRR.ugL**	Concentration of cyanobacterial hepatotoxin microcystin dmRR	Micrograms per litre
**MC_RR_ugL**	Concentration of cyanobacterial hepatotoxin microcystin RR	Micrograms per litre
**MC_dmLR_ugL**	Concentration of cyanobacterial hepatotoxin microcystin dmLR	Micrograms per litre
**MC_LR_ugL**	Concentration of cyanobacterial hepatotoxin microcystin LR	Micrograms per litre
**MC_LY_ugL**	Concentration of cyanobacterial hepatotoxin microcystin LY	Micrograms per litre
**MC_LW_ugL**	Concentration of cyanobacterial hepatotoxin microcystin LW	Micrograms per litre
**MC_LF_ugL**	Concentration of cyanobacterial hepatotoxin microcystin LF	Micrograms per litre
**NOD_ugL**	Concentration of cyanobacterial hepatotoxin nodularin	Micrograms per litre
**CYN_ugL**	Concentration of cyanobacterial cytotoxin cylindrospermopsin	Micrograms per litre
**ATX_ugL**	Concentration of cyanobacterial neurotoxin anatoxin-a	Micrograms per litre
